# Meta-transcriptomic characterization reveals viral species with zoonotic potential in *Rhipicephalus microplus* and *Haemaphysalis bispinosa* ticks in Pakistan

**DOI:** 10.1186/s13567-026-01739-5

**Published:** 2026-03-26

**Authors:** Laila Jamil, Yu-Yu Li, Cheng Li, Nuo Cheng, Yi-Fei Wang, Jabran Jamil, Ning Wang, Wan-Nian Wei, Yi Sun, Biao Deng, Abakundana Nsenga Ariston Gabriel, Run-Ze Ye, Wu-Chun Cao, Lin Zhao

**Affiliations:** 1 Institute of EcoHealth, School of Public Health, Shandong University, Jinan, Shandong People’s Republic of China; 2 State Key Laboratory of Pathogen and Biosecurity, Academy of Military Medical Sciences, Beijing, People’s Republic of China; 3 Institute of Pathogen Biology, Chinese Academy of Medical Sciences & Peking Union Medical College, Beijing, China; 4 Livestock and Dairy Development Department (Ext) Peshawar, Khyber Pakhtunkhwa, Pakistan; 5 Department of Emergency Medicine, Qilu Hospital of Shandong University, Jinan, Shandong People’s Republic of China; 6 Shandong Provincial Key Laboratory of Intelligent Monitoring, Early Warning, Prevention and Control for Infectious Diseases, Jinan, China

**Keywords:** Ticks, virome, evolution, virus diversity, Pakistan

## Abstract

Tick-borne viruses are an increasing global health concern, but the tick virome in South Asia, particularly in Pakistan, remains poorly underexplored. Pakistan's subtropical climate and agricultural environment provide favorable habitats for ticks, making it an endemic region for various tick-borne pathogens. This study aimed to characterize the tick-associated virome and viral diversity of ticks in Khyber Pakhtunkhwa Province, Pakistan, and assess the potential risks to human and livestock health. A meta-transcriptomic investigation was conducted on host seeking adult ticks collected from livestock at 19 sites across the province. A total of 23 pooled samples, consisting of 165 individual ticks of *Rhipicephalus microplus* and *Haemaphysalis bispinosa*, were analyzed. The viral characterization involved diversity assessment and phylogenetic analysis, identifying ten RNA virus species from nine families, including two previously undefined novel viruses, below the ICTV species demarcation thresholds. Notably, Mogiana tick virus has been reported to be pathogenic to humans, whereas *Pakistan microplus* virus and *Uukuvirus lihanense* are phylogenetically closely related to viruses infecting humans, suggesting a potential risk of cross-species transmission to both humans and livestock. This study provides the first comprehensive dataset of the tick virome in Pakistan, highlighting the need for enhanced surveillance of tick-borne viral infections in humans and livestock.

**Supplementary Information:**

The online version contains supplementary material available at 10.1186/s13567-026-01739-5.

## Introduction

Ticks (*Acari: Ixodida*) are obligate, haematophagous arthropod vectors transmitting a wide range of viral, bacterial and protozoan pathogens to humans, domestic and wild animals. Owing to the rapid development of metagenomic sequencing technology, a plethora of previously undefined viruses were recently identified in various ticks around the world [1–3]. More than 700 viral species have been reported across 31 tick species in China [1], and 19 novel viruses were discovered in just 146 ticks from two locations in Australia [2]. These findings highlight both the high diversity in tick virome and our insufficient understanding of tick-associated viruses, considering the total number of tick species and their extensive global distribution. Therefore, investigating tick virome in different regions is necessary to better understand the global viral sphere of ticks and their relevance to public health [4].

Pakistan is located in the subtropical zone, and is an agricultural country where animal husbandry is one of the major components of the national economy [5], and the climatic and ecological environments provide favorable habitats for ticks. Over two-thirds of the population of Pakistan live in rural areas, with most relying on livestock for their livelihood. A recent surveillance study revealed that patients with tick-borne *Crimean-Congo hemorrhagic fever* (CCHF) have been reported in all provinces of Pakistan with a fatality rate about 25% [6], making CCHF, one of the most lethal infectious diseases of humans. Meanwhile, tick-borne diseases in livestock have caused huge economic losses due to substantial morbidity and mortality with reduced production [7, 8]. However, the burden of tick-borne diseases in humans and domestic animals are poorly understood, and tick virome has never been investigated in Pakistan. In this study, we conducted a meta-transcriptomic analysis of ticks collected in a pasturing region of Pakistan to understand the tick-associated virus profile, identify the potential viral pathogens to humans and animals, and ultimately provide fundamental data for making prevention and control strategies for tick-borne viral infections. Khyber Pakhtunkhwa was prioritized due to its high livestock density and documented cases of Crimean-*Congo hemorrhagic fever*, which poses significant public health risks.

## Materials and methods

### Collection of ticks

**Figure 1:**
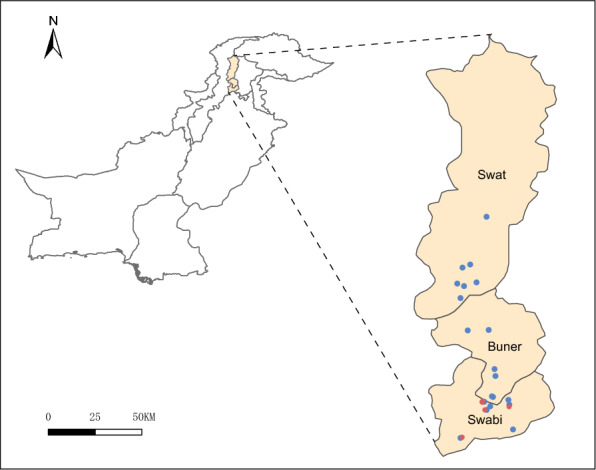
**Geographic distribution of tick samples used for the meta-transcriptomic sequencing**. Solid blue circles represent the collections sites of *Rhipicephalus microplus,* while Solid red circles represent the collections sites of *Haemaphysalis bispinosa*.

A total of 165 ticks were collected from three districts (Swat, Buner and Swabi) in a region with agriculture and grazing lands of Khyber Pakhtunkhwa Province, Pakistan from “December 2023 to March 2024”. Ticks were collected from livestock included Bos taurus (cattle), Ovis aries (sheep), and Capra hircus (goats), across 19 sites, based on both ecological and methodological considerations (Figure 1). No ethical approval required as per local regulations. The species, sex and developmental stage of each tick were first identified by an entomologist (Yi Sun). The latitude and longitude of each collection site were recorded while the ticks were collected (Additional file 1). Host-questing adult ticks were divided into pools based on species, sex, and sampling sites.

### RNA extraction, library preparation, and sequencing

After surface sterilization (two successive 30-s washes in 70% ethanol), total RNA was extracted from each pool (n = 23) using the AllPrep DNA/RNA mini kit (Qiagen). Negative extraction and sequencing controls (NTCs) were not included in this study, their inclusion is recommended for future research. The number of ticks per pool varied intentionally, with an average of approximately 7 ticks per pool. Briefly, ticks were homogenized in RLT (Ribonucleic Acid Lysis) solution in a mortar treated with liquid nitrogen. Future studies should include the analysis of individual ticks to detect rare or low-abundance viruses. The homogenate was then incubated with proteinase K (Qiagen) at 55 °C for 10 min and centrifuged at 15 000 *g* for 30 s. The homogenized lysate was transferred to an AllPrep DNA spin column and centrifuged at 8000 *g* for 30 s. The flow-through was used for RNA purification following the manufacturer’s instructions.

The purified RNA was quantified using Qubit 4.0 fluorometer (Thermo Fisher Scientific, United States), and RNA quality was assessed using an Agilent Bioanalyzer 2200 (Agilent Technologies, United States). The ribosomal RNA was removed using RiBo-Zero Gold rRNA removal reagents (Human/Mouse/Rat) (Illumina). Then, the sequencing library was prepared following the Illumina standard protocol. RNA input was normalized across pools to ensure consistency in library preparation. Paired-end (2 × 150 bp) transcriptome sequencing (RNA-seq) was conducted on an Illumina HiSeq 6000 platform at Novogene Tech (Beijing, China).

### Virome diversity analysis

Subsequent analysis followed the tick virome protocols [9]. The AfterQC (V2.3.3) [10] was employed to filter raw sequencing reads by removing those with low quality (Phred score < 15) and short read lengths (< 35 bp). The clean reads were mapped against the *Rhipicephalus microplus* (GCF_013339725.1 BIME_Rmic_1.3) (2022.08.22) and related tick genome *Haemaphysalis longicornis* genome (GCA_013339765.1 BIME_HaeL_1.3) (2021.12.07) using Bowtie2 (v2.3.5.1) with default parameters [11] to remove reads associated with the ticks. The remaining reads were compared to the NCBI nucleotide sequence database by Kraken2 (v2.1.2) [12], and the virus abundance of each family was measured as the number of mapped reads per million viral reads in the library. Alpha diversity was measured at viral family level using the Shannon index, with calculations performed using the Rhea alpha diversity script [13] in the R software. Bray–Curtis PCoA was performed on viral family-level read abundances. Based on the Bray–Curtis distance index, the similarity matrix was calculated for different groups, and principal coordinates analysis was used to analyze the β-diversity of virome among different tick groups. Statistical differences in α-diversity among groups of different tick species, gender, or host were assessed by the Wilcoxon rank-sum test. Differences among sampling cities (three groups) were assessed by first testing for normality with the Shapiro–Wilk test and for homogeneity of variances with Levene’s test. When both parametric assumptions were satisfied, one-way ANOVA was conducted, followed by Tukey’s post hoc test for pairwise comparisons with p-values adjusted for multiple testing. If assumptions were not met, the non-parametric Kruskal–Wallis test was applied, with subsequent post hoc pairwise comparisons. All statistical analyses were performed in R software. Future studies should include a power analysis to ensure sufficient sensitivity for detecting differences in diversity.

### Transcriptome analysis and virus annotation

Quality-controlled viral reads were de novo assembled using Trinity (v2.13.2) [14]. All assembled contigs were compared against the NCBI nonredundant protein database (nr, 2023/3/12) using Diamond BLASTx (v2.0.13) [15] and against the NCBI nucleotide sequence database (nt, 2023/3/04) using blastn (v2.12.0 +) [16] to identify virus-associated contigs based on the top BLAST hits of each contig. The E-value threshold for significant hits was set to 1 × 10^−5^, as mentioned in the methods section. The potential open reading frames (ORFs) of viral contigs were compared with reference sequences in NCBI using Geneious prime v2025.0.3 with default parameters. The conserved RNA-dependent RNA polymerase (RdRP) domain and capsid protein were annotated by comparing to the Conserved Domain Database [17].

### Virus classification

The viruses identified in this study were classified according to the species demarcation criteria issued by the International Committee on Taxonomy of Viruses (ICTV). In case such criteria were unavailable in ICTV website, a putative novel virus was proposed if nucleotide (nt) identity across the whole viral genome was < 80% or amino acid (aa) similarity of its RdRP domain was < 90% in comparison to all known viruses as previously described [18, 19]. The putative novel viruses were provisionally denominated as “Pakistan”, plus the virus-related genus name or the family name (excluding “viridae” characters). Moreover, the new viral genome was confirmed by checking read coverage and continuity using Bowtie2. The mapping statistics for the putative novel viruses identified in this study include details such as virus species, library ID, accession numbers, open reading frame (ORF) lengths, mapped reads, mean read depth, and genome coverage percentages s (Additional file 2).

### Phylogenetic analysis

The aa of RdRP domain in viral sequences were aligned with related references downloaded from GenBank using MAFFT (v7.490) [20] and ambiguously aligned regions were removed using TrimAl (v1.4) [21]. Maximum likelihood (ML) phylogenetic trees were constructed using IQ-TREE (v2.2.2.3) algorithm [22] with 1000 bootstrap replicates based on the sequence alignments with Model Finder selected for optimal model selection. The trees were visualized using suitable packages in R. For display purposes, the trees were unrooted using the midpoint rooting method, however, they remain fundamentally unrooted.

## Results

### Diversity in tick virome

A meta-transcriptomic investigation on tick virome was conducted in the Khyber Pakhtunkhwa Province of Pakistan, with samples collected from Buner (*n* = 7), Swabi (*n* = 8), and Swat (*n* = 8). Host-questing adult ticks were collected from Swabi, Buner, and Swat districts from “December 2023 to March 2024” (Figure 1) and pooled according to collection site, species, and sex, with each pool consisting of 4–10 ticks (Additional file 3). As a result, a total of 23 libraries were successfully constructed for meta-transcriptome sequencing, including 19 pools of *Rhipicephalus microplus* and 4 pools of *Haemaphysalis bispinosa*, yielding 9.1 × 10⁸ paired-end reads of 100–150 bp in length. After quality control and removal of host genomic reads, 2.1 × 10⁶ viral reads were identified, accounting for 0.55% of the total clean reads. The percentage of viral reads per library ranged from 0.01% to 17.0%, with an interquartile range (IQR) of 0.07%–0.15% (Additional file 3).

**Figure 2:**
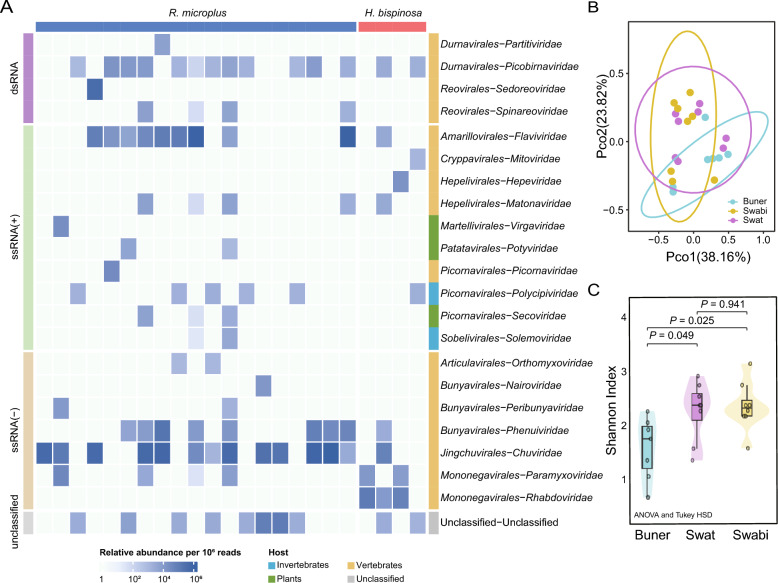
**Virome diversity *Rhipicephalus microplus* and *Haemaphysalis bispinosa* ticks.** **A** Relative abundance of viral families detected in each library. Each cell in the heat map represents the normalized number of reads belonging to the viral family. **B** Between-group clustering of viromes among three districts by Principal Coordinate Analysis of Bray–Curtis dissimilarities, with axes 1 (38.16%) and 2 (23.82%). **C** Comparison of the viral Shannon Index among three districts. Boxplot elements: center line, median; box limits, upper and lower quartiles; whiskers (error bars), the highest and lowest points within the 1.5 interquartile range of the upper and lower quartiles. Statistical significance between groups was determined using one-way ANOVA (overall *P* = 0.0199). Exact *P*-values for diversity comparisons: *P* = 0.283 for *R. microplus* vs *H. bispinosa*. Adjusted *p*-values are shown above the comparison brackets.

Viral reads were classified into 29 established viral families and a group of unclassified viruses after removing bacteriophages through comparison with the NCBI nucleotide sequence database. Among these, 20 families were associated with vertebrates, each exhibiting distinct detection frequency and abundance patterns (Figure 2A). The most frequently detected vertebrate-infecting viruses belonged to *Chuviridae* (order *Jingchuvirales*), *Phenuiviridae* (order *Bunyavirales*), and *Flaviviridae* (order *Amarillovirales*), showing relatively high abundance and detection frequency both in *R. microplus* and *H. bispinosa*. Unclassified viral reads were additionally detected in most tick samples.

Viral operational taxonomic units (OTUs) estimated from meta-transcriptomic data revealed regional differences in virome diversity. Principal Coordinates Analysis demonstrated distinct clustering patterns among ticks collected from different geographical regions (Figure 2B). Shannon index analysis of α-diversity indicated significantly lower viral diversity in Buner compared to Swabi (P < 0.05) and Swat (P < 0.05), whereas no significant difference existed between Swabi and Swat districts (Figure 2C). No notable variations in viral diversity were observed across tick species, sexes, or host associations (Additional file 4). These regional differences in virome diversity were observed under the current sampling design and time window, and potential influences of sampling conditions cannot be completely excluded.

### Assembly of viral genomes and taxonomy

**Figure 3:**
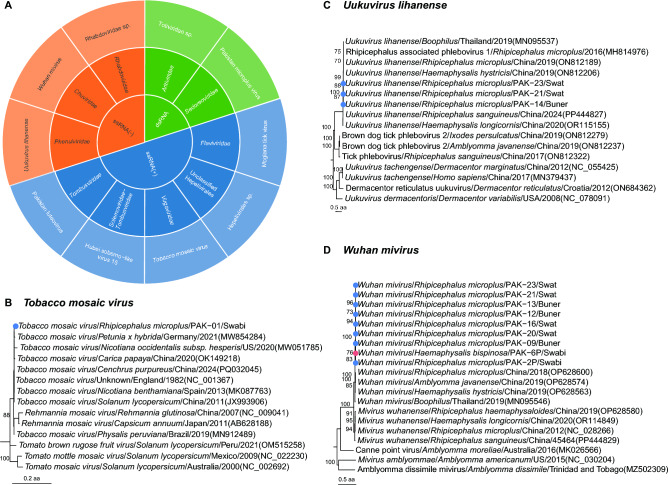
**Phylogenetic analysis of viruses approved by the International Committee on Taxonomy of Viruses.** **A** Taxonomic distribution of RNA viruses identified in this study. **B** Phylogeny of viruses in the species *Tobacco mosaic virus* of the family *Virgaviridae* based on RdRP domain. **C** Phylogeny of viruses in the species *Uukuvirus lihanense* of the family *Phenuiviridae* based on RdRP domain. **D** Phylogeny of viruses in the species *Wuhan mivirus* of the family *Chuviridae* based on RdRP domain. Viruses of different tick species detected in this study are represented by different colors.

Following de novo assembly, 27 905 viral contigs were obtained, from which 58 RNA virus genomes were assembled and deposited in GenBank (Additional file 5). Viruses were classified according to the taxonomy framework established by ICTV. This study identified 10 viral species spanning 9 families and 1 unclassified family across 9 orders and 1 unclassified order, including 2 dsRNA viruses, 3 ssRNA(+) viruses, and 5 ssRNA(-) viruses (Figure 3A). Among them, three viruses were known species approved by ICTV, including *Tobacco mosaic virus* (TMV), *Uukuvirus lihanense*, and *Wuhan mivirus*. Five viruses, including Totiviridae sp., Mogiana tick virus, Hepelivirales sp., Hubei sobemo − like virus 15, and Rhabdoviridae sp. had been previously reported viruses, but had not been approved by ICTV. The remaining two viruses in the family *Sedoreoviridae* and *Tombusviridae* were genetically divergent from any known viral species and proposed as undefined viral species, provisionally designated as *Pakistan microplus* virus and Pakistan luteovirus according to their phylogenetic position and original country and tick species (Table 1).

**Table 1:** **Viruses identified in the present study**.

Tick species	District	Virus (Strain)	Closet relative viruses	aa (%)^a^	nt (%)^b^
*R. microplus*	Swabi	*Tobacco mosaic virus* (PAK-01)	*Tobacco mosaic virus*	100	99.7
*R. microplus*	Swat	*Uukuvirus lihanense* (PAK-23)	*Uukuvirus lihanense*	98.7	93.5
*R. microplus*	Swat	*Uukuvirus lihanense* (PAK-21)	*Uukuvirus lihanense*	98.7	93.4
*R. microplus*	Buner	*Uukuvirus lihanense* (PAK-14)	*Uukuvirus lihanense*	98.6	93.4
*R. microplus*	Swat	*Wuhan mivirus* (PAK-23)	*Wuhan mivirus*	97.3	92.8
*R. microplus*	Swat	*Wuhan mivirus* (PAK-21)	*Wuhan mivirus*	97.4	92.7
*R. microplus*	Swat	*Wuhan mivirus* (PAK-20)	*Wuhan mivirus*	97	92.6
*R. microplus*	Swat	*Wuhan mivirus* (PAK-16)	*Wuhan mivirus*	97.1	92.7
*R. microplus*	Buner	*Wuhan mivirus* (PAK-13)	*Wuhan mivirus*	97.4	92.8
*R. microplus*	Buner	*Wuhan mivirus* (PAK-12)	*Wuhan mivirus*	97.3	92.8
*R. microplus*	Buner	*Wuhan mivirus* (PAK-09)	*Wuhan mivirus*	95.3	91.5
*R. microplus*	Swabi	*Wuhan mivirus* (PAK-02)	*Wuhan mivirus*	97.3	92.8
*R. microplus*	Swabi	*Wuhan mivirus* (PAK-01)	*Wuhan mivirus*	89.8	93.7
*H. bispinosa*	Swabi	*Wuhan mivirus* (PAK-06)	*Wuhan mivirus*	97.1	92.7
*R. microplus*	Swat	Totiviridae sp. (PAK-23)	Totiviridae sp.	92.8	84.1
*R. microplus*	Swat	Totiviridae sp. (PAK-16)	Totiviridae sp.	93	90.3
*R. microplus*	Buner	Totiviridae sp. (PAK-12)	Totiviridae sp.	99.1	94.8
*R. microplus*	Swat	Mogiana tick virus (PAK-23)	Mogiana tick virus	98.1	93.8
*R. microplus*	Swat	Mogiana tick virus (PAK-21)	Mogiana tick virus	92	93.7
*R. microplus*	Swat	Mogiana tick virus (PAK-20)	Mogiana tick virus	81	92.9
*R. microplus*	Swat	Mogiana tick virus (PAK-16)	Mogiana tick virus	91.7	94.6
*R. microplus*	Buner	Mogiana tick virus (PAK-14)	Mogiana tick virus	97.6	94.2
*H. bispinosa*	Swabi	Hepelivirales sp. (PAK-07)	Hepelivirales sp.	94.4	84.8
*H. bispinosa*	Swabi	Hubei sobemo-like virus 15 (PAK-06)	Hubei sobemo-like virus 15	93	88.1
*R. microplus*	Swat	Rhabdoviridae sp. (PAK-20)	Rhabdoviridae sp.	96.4	83
*R. microplus*	Swat	Pakistan microplus virus (PAK-16)	*St Croix River virus*	86.9	76.6
*R. microplus*	Buner	Pakistan luteovirus (PAK-10)	Cheeloo luteovirus 1	82.2	77.8
*R. microplus*	Swabi	Pakistan luteovirus (PAK-04)	Cheeloo luteovirus 1	82.4	78.4

a: amino acid identity of conserved RNA-dependent RNA polymerase.

b: nucleotide identity of genome.

### Viruses approved by ICTV

Among ICTV-approved viruses, TMV, a ssRNA(+) in the family *Virgaviridae*, was detected in one *R*. *microplus* pool from the Swabi district. Phylogenetic analysis based on the RdRP domain revealed that these viruses formed a distinct branch with Totiviridae sp. infecting *R. microplus* in Yunnan, China (GenBank accession number ON812585), sharing 92.8% to 99.1% amino acid identity in the RdRP domain with that of TMV standard strain (GenBank accession number NC_001367) from UK (Figure 3B). TMV is a well-known pathogen causing severe damage to tobacco and other crops, including tomatoes, papayas, and petunias, leading to stunted plant growth and reduced yields [23–25]. Notably, we assembled its whole viral genome, which possessed a nt identity of 99.7% with the UK standard TMV strain (Table 1). The 100% amino acid and 99.7% nucleotide identity of TMV detected in this study likely resulted from passive acquisition through contact with infected plant material, as ticks are not known to transmit plant viruses. Another virus approved by ICTV was *Uukuvirus lihanense* in the family *Phenuiviridae* of order *Bunyavirales*, which was detected in three *R. microplus* pools from Swat and Buner districts, showing 99.7%−100% aa identity of RdRP domain with each other and 98.6%−98.7% to the viral sequence from *R. microplus* (GenBank accession number ON812189) in Chongqing, China (Figure 3C). The third was *Wuhan mivirus* in the family *Chuviridae* of order *Jingchuvirales*, which exhibited the highest positive rate across all viruses, identified in 39.1% (9/23) of samples from both *R. microplus* (42.1%, 8/19) and *H. bispinosa* (25%, 1/4) in all three districts. These viruses displayed aa identity of RdRP domain ranging from 91.4% to 100%, and showed 89.8%–97.4% aa identities with that of the known strain detected in *R. microplus* in Sichuan, China (GenBank accession number OP628600). Phylogenetic analysis revealed that *Wuhan mivirus* in this study clustered with global variants detected in diverse tick species, indicating its broad geographic and vector distribution (Figure 3D).

### Known viruses not approved by ICTV

**Figure 4:**
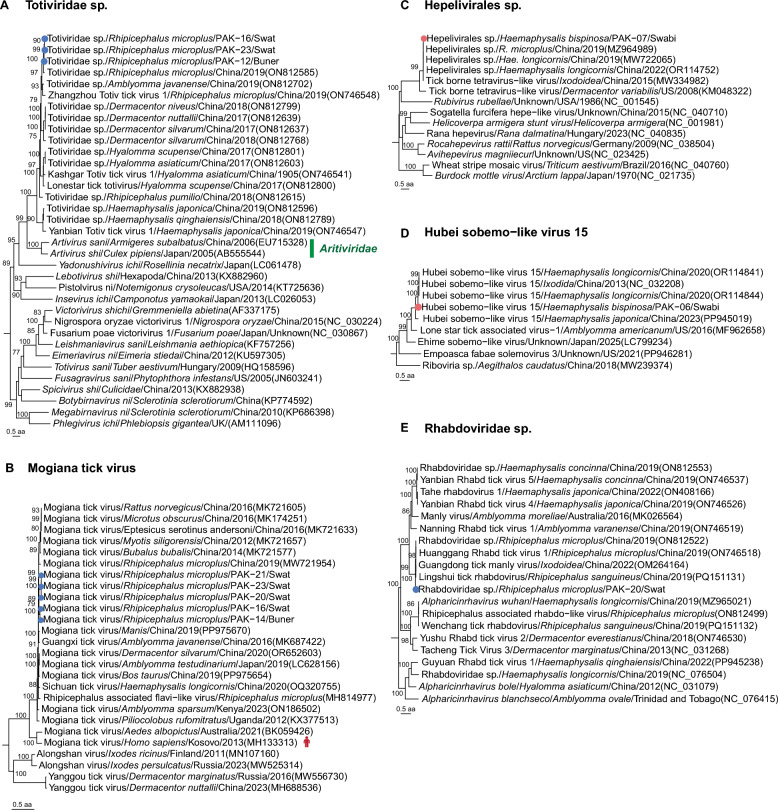
**Phylogenetic analysis of known viruses not approved by ICTV.** **A** Phylogeny of viruses in the species Totiviridae sp. based on RdRP domain. **B** Phylogeny of viruses in the species Mogiana tick virus based on RdRP domain. **C** Phylogeny of viruses in the species Hepelivirales sp. based on RdRP domain. **D** Phylogeny of viruses in the species Hubei sobemo − like virus 15 based on RdRP domain. **E** Phylogeny of viruses in the species Rhabdoviridae sp. based on RdRP domain. ICTV, the International Committee on Taxonomy of Viruses.

Among previously reported viruses but not approved by ICTV, Totiviridae sp., a dsRNA from the order *Ghabrivirales*, was identified in *R. microplus* ticks collected from Swat and Buner districts. Phylogenetic analysis based on the RdRP domain revealed that these viruses formed a distinct branch with Totiviridae sp. infecting *R. microplus* in Yunnan, China (GenBank accession number ON812585), sharing 92.8%–99.1% aa identity of RdRP domain. This branch exhibited close evolutionary relationships with viruses in the family *Artiviridae* (Figure 4A). Mogiana tick virus, a ssRNA(+) virus in the family *Flaviviridae* of order *Amarillovirales*, was detected in *R. microplus* ticks from Swat and Buner districts. Phylogenetic analysis revealed its closest relationship to Mogiana tick virus identified in *Amblyomma testudinarium* from Ozu, Japan (GenBank accession number LC628156), with 81.0%−98.1% aa similarity of RdRP (Figure 4B). Studies indicate this virus can be transmitted by multiple tick species and infect diverse hosts, including bats, rodents, cattle, and primates. The zoonotic risk of Mogiana tick virus (Jingmen tick virus) is identified in human patients with *Crimean-Congo hemorrhagic fever* in Kosovo [26]. This raises concerns about the potential for similar human infections through tick bites, highlighting the importance of further investigation. Another ssRNA(+) virus, Hepelivirales sp. (order *Hepelivirales*), was detected in a *H. bispinosa* from Swabi district. It was genetically closest to Hepelivirales sp. found in *H. longicornis* from Shandong, China (GenBank accession number OR114752), and sharing 94.4% RdRP aa identity (Figure 4C). The ssRNA(+) Hubei sobemo − like virus 15 was a virus, positioned phylogenetically between the families *Solemoviridae* and *Tombusviridae*. Phylogenetic analysis based on the RdRP domain revealed the closest genetic proximity to a virus identified in *H. longicornis* from Shandong, China, with 93.0% aa identity (Figure 4D) [19]. A ssRNA(-) virus called Rhabdoviridae sp. in the order *Mononegavirales* was identified in *R. microplus* from Swat district, sharing 96.4% aa similarity of RdRP domain with that of the virus from *R. microplus* (GenBank accession number ON812522) in China (Figure 4E).

### Undefined viruses identified in this study

**Figure 5:**
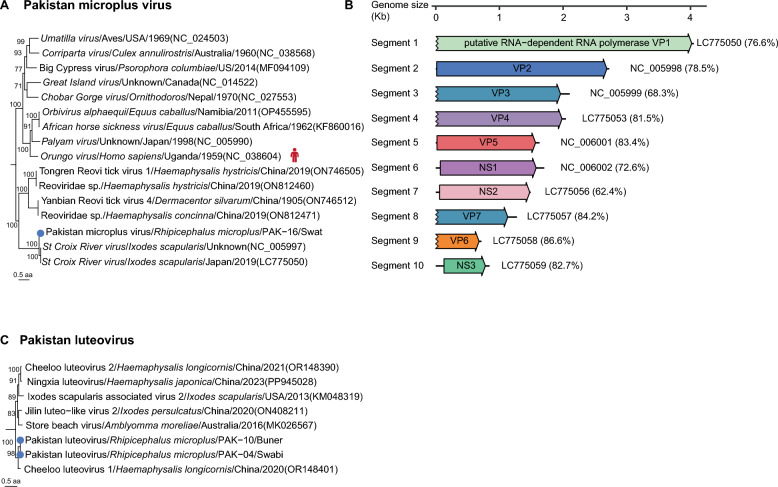
**Phylogenetic analysis of undefined viruses.** **A** Phylogeny of viruses in the species *Pakistan microplus* virus based on RdRP domain. **B** Genome structure of *Pakistan microplus* virus. The genomes are drawn to a unified length scale. The right side of the genome corresponds to the nearest nucleic acid sequence, with the nucleotide similarity in parentheses. **C** Phylogeny of viruses in the species Pakistan luteovirus based on RdRP domain.

Two undefined viruses, provisionally denominated as *Pakistan microplus* virus and Pakistan luteovirus, were identified in this study. *Pakistan microplus* virus, a dsRNA virus in the genus *Orbivirus* of family *Sedoreoviridae*, was detected in *R. microplus* ticks from Swat district. Phylogenetic analysis based on the RdRP domain revealed it was clustered with *St. Croix River virus* isolated from the *Ixodes scapularis* IDE8 cell line in Japan (GenBank accession number LC775050) [27], with 86.9% aa identities of RdRP domain (Figure 5A). Notably, all the ten segments of *Pakistan microplus* virus were assembled, which had variable nt similarities with those of *St. Croix River virus*, further proving the virus as a novel species (Additional file 6 and 7). Of significant concern, *Orungo virus*, a ten-segmented congeneric virus reported in Uganda in 1959, has demonstrated human infectivity [28]. These findings suggest potential zoonotic spillover risks for the *Pakistan microplus* virus. Enhanced surveillance and research on such emerging pathogens could shift public health strategies from passive defense to proactive prevention and control measures.

Pakistan luteovirus, being a ssRNA(+) virus in the genus *Luteovirus* of the family *Tombusviridae*, was detected in *R. microplus* ticks from the Buner and Swabi districts. Phylogenetic analysis based on the RdRP domain revealed its clustering with Cheeloo luteovirus 1 identified in *H. longicornis* ticks from Shandong, China (GenBank accession number OR148401), sharing 82.2%−82.4% aa homology [19]. While, the two Pakistan luteovirus strains from Buner and Swabi exhibited 99.8% aa homology of RdRP domain (Figure 5C). The emergence of such viruses may result from cross-species transmission among tick populations, geographic dispersal, and long-term adaptive evolution.

## Discussion

Pakistan is an agricultural country, where animal husbandry constitutes a major component of its national economy [5], and the climate and ecological environment provide favorable habitats for ticks. However, no virome investigation has been conducted in Pakistan to date. This study collected ticks across 19 sites in three districts of Khyber Pakhtunkhwa Province, Pakistan to explore the viral spectrum carried by local ticks. Among the 23 samples analyzed (19 *R*. *microplus* and 4 *H*. *bispinosa*), 10 viruses from 9 families and one unclassified viral family were identified, including 3 ICTV-recognized viruses, 5 known tick-associated viruses not yet accepted by ICTV, and 2 novel viruses previously undefined. Six viruses, including *Uukuvirus lihanense* and *Wuhan mivirus* (officially approved by ICTV), as well as Totiviridae sp., Mogiana tick virus, Hepelivirales sp., and Hubei sobemo − like virus 15 (known but not yet ICTV-accepted) exhibited relatively high detection rates, which have been detected among multiple tick species. *Wuhan mivirus*, Hepelivirales sp., and Hubei sobemo-like virus 15 were first detected in *H*. *bispinosa* ticks. These findings suggest that these viruses have evolved into generalists within tick populations to better tolerate or adapt to environmental changes [1]. Notably, Mogiana tick virus is not only carried by multiple tick species but also infects diverse reservoir hosts such as bats, rodents, cattle, and monkeys, completing the virus-vector-host transmission cycle. While Mogiana tick virus (Jingmen tick virus) has been found in ticks and cattle in Brazil, its potential to infect humans raises concern. It reported association with human cases of *Crimean-Congo hemorrhagic fever* in Kosovo, underscores the need for further investigation, highlighting its potential risk to human populations [26, 29].

In this virome study, we identified two previously undefined viruses including *Pakistan microplus* virus and Pakistan luteovirus. The *Pakistan microplus* virus, detected in the Swat district, belongs to the genus *Orbivirus*, which can be transmitted by various vectors including ticks and mosquitoes, and infect diverse mammals. *Orungo virus*, which belongs to the same viral genus, has been extensively documented in humans, livestock, wildlife, and arthropod vectors [30]. Another undefined virus, Pakistan luteovirus, is distinctive from all known luteoviruses that have been identified in multiple tick species from different countries, suggesting complex genetic evolutionary relationships between these virus species in various tick vectors and their geographical origins. The emergence of novel viruses may result from cross-species transmission among ticks and geographical dispersal. These findings underscore the necessity for enhanced human infection surveillance, particularly in areas inhabited by *R*. *microplus* ticks, to enable early detection and targeted prevention of emerging tick-borne diseases, thereby minimizing public health threats. On the other hand, these findings also indicate that viromic study in ticks can facilitate the discovery of potential pathogenic viruses from an etiological perspective [31], which may pose threats to public and veterinary health [4].

Strikingly, TMV, well-known as a plant pathogen that severely damages tobacco, tomatoes, and other crops [23–25], was first detected in *R. microplus* ticks in Pakistan. The detection of TMV exclusively in the Swabi rather than in the other two districts might be related to environmental or ecological factors, with Swabi district being the primary tobacco-growing area in Khyber Pakhtunkhwa Province of Pakistan, although the underlying reasons remain unclear [32]. The detection of TMV indicates possible atypical exposure to a plant virus, but does not support the conclusion that ticks can transmit this virus. Given that ticks are obligate blood-feeding arthropods and no experimental evidence currently supports their ability to carry this virus or infect non-plant organisms (arthropods or mammals), the mechanism of TMV acquisition by *R. microplus* requires further investigation. The tick might have passively acquired the virus through contact with TMV-infected plants. Regardless of the acquisition route, the finding indicates that ticks might harbor, disseminate, or mechanically transmit diverse viruses, underscoring the critical need for tick virome studies to map their viral repertoires. Recent virome analyses have shown that ticks, including *Haemaphysalis bispinosa*, can harbor plant viruses such as *Turnip mosaic virus* and *Turnip yellows virus* along with a broad diversity of RNA viruses from viral families associated not only with vertebrates but also with fungi and plants, suggesting that ticks might acquire these viruses through passive transmission from environmental sources [1, 33]. Furthermore, the presence of TMV in *R. microplus* highlights the need for further investigation into unconventional arbovirus transmission pathways.

One limitation of this study is the absence of extraction and sequencing negative controls, which are essential for distinguishing true viral signals from contamination in meta-transcriptomic workflows. This is especially relevant for low abundance viruses, like TMV and Pakistan microplus virus, or those detected in a single library. Although standard procedures were followed, low abundance viral reads were carefully checked for coverage and continuity. Future studies should incorporate negative controls during extraction and library preparation.

Another limitation is the pooling of multiple ticks from the same species, sex, and sampling location into a single library, which prevented virus detection at the individual tick level and may have reduced sensitivity for rare viruses. Pooled samples also do not accurately reflect true virus prevalence within tick populations. Sampling was conducted from “December 2023 to March 2024”, representing winter and early spring, and therefore does not account for seasonal variation in tick activity or virome composition. Future work should incorporate multi-seasonal and longitudinal sampling. Importantly, these limitations are unlikely to affect viruses consistently detected across multiple pools and districts, which showed coherent genomic structure and phylogenetic placement.

In summary, this study represents the first viromic investigation of ticks in Pakistan, demonstrating the viral diversity carried by *R. microplus* and *H. bispinosa* and revealing the composition of viruses in the surveyed districts. These findings fill a critical knowledge gap in tick virome research within Pakistan. The current outcomes establish a new foundation for further research on virus-vector-host interactions and ultimately assessing the risk of viral transmission from *R. microplus* and *H. bispinosa* to humans and animals.

## Conclusions

This study represents the first characterization of the tick virome in Pakistan, providing a comprehensive dataset of viruses carried by local tick species. We identified two novel viral species along with multiple known viruses, thereby expanding the global repository of tick-associated viral diversity.

Notably, among the ten viruses detected, *Pakistan microplus* virus and *Uukuvirus lihanense* may represent potential zoonotic threats, while Mogiana tick virus has documented human infections. The detection of Mogiana tick virus on cattle and sheep in the geographically adjacent Swat and Buner districts highlights the potential risk of zoonotic transmission in these regions. The findings highlight the need for proactive surveillance of tick-borne diseases in both livestock and humans, which would enable early detection and targeted prevention strategies within these districts and surrounding areas. This underscores the importance of shifting public health and veterinary strategies from passive defense to proactive prevention in both human and animal health. Furthermore, they emphasize the critical need for enhanced tick monitoring across Pakistan and the broader South Asian region.

## Supplementary Information

**Additional file 1**. **Basic information of each site for sampling in this study.**

**Additional file 2**. **Mapping Statistics for putative novel viruses identified in this study.**

**Additional file 3**. **Basic information of each library for sequencing in this study.**

**Additional file 4**. **Virome diversity across tick species, sex, and host.**

**Additional file 5**. **Viral sequences of this study deposited in GenBank.**

**Additional file 6**. **Phylogenetic tree of orbivirus strains showing their genetic relationships across various hosts and region.**

**Additional file 7**. **Phylogenetic tree of orbivirus strains based on VP7, NS1, NS2, and NS3 protein sequences, illustrating their molecular relationships.**

## Data Availability

Not applicable. This study did not involve human participants or animals.

## Abbreviations

ICTV: International Committee on Taxonomy of Viruses
CCHF: *Crimean-Congo hemorrhagic fever*
ORFs: open reading frames
RdRP: RNA-dependent RNA polymerase
OTUs: operational taxonomic units
TMV: *Tobacco mosaic virus*

## Declarations

### Availibility of data and materials

All datasets have been included with this article and our sequences have been deposited within GenBank (accession number PV129914‐PV167521, Additional file 5).
