# Supplementary material for: Meta-transcriptomic characterization reveals viral species with zoonotic potential in Rhipicephalus microplus and Haemaphysalis bispinosa ticks in Pakistan
Source: Vet Res. 2026 Mar 26;57:56. doi: 10.1186/s13567-026-01739-5 (PMC13107747; doi:10.1186/s13567-026-01739-5)
Supplement: Supplementary file 1 — Additional file 1. Basic information of each site for sampling in this study. [file 13567_2026_1739_MOESM1_ESM.docx]

**Additional file 1**. Basic information of each site for sampling in this study

| District | Site name | Latitude (°N) | Longitude (°E) | Dominant land-use description |
| --- | --- | --- | --- | --- |
| Swabi | Maneri Payan | 34.1445 | 72.4909 | Intensive irrigated agriculture and peri-urban livestock farming |
| Swabi | Swabi | 34.1270 | 72.4741 | Mixed cropland with dense livestock holdings |
| Swabi | Saleem Khan Khel | 34.1664 | 72.4621 | Irrigated farmland dominated by tobacco cultivation |
| Swabi | Batakara | 34.0338 | 72.6003 | Rural mixed farming and open grazing |
| Swabi | Jhanda | 34.1523 | 72.5829 | Agricultural plains with small-scale dairy farming |
| Swabi | Beka | 33.9925 | 72.3483 | Cropland with seasonal grazing |
| Swabi | Panjman | 34.1749 | 72.5782 | Rain-fed agriculture and communal grazing |
| Buner | Totalai | 34.1923 | 72.4990 | Hilly subsistence agriculture with livestock rearing |
| Buner | Dagai | 34.1892 | 72.5043 | Rural village agriculture with livestock sheds |
| Buner | Kas Koruna | 34.5083 | 72.3835 | Mountain foothills with extensive grazing |
| Buner | Chinglai | 34.3227 | 72.5116 | Pastoral grazing and low-intensity agriculture |
| Buner | Sawawai | 34.2897 | 72.5160 | Rain-fed agriculture and hillside grazing |
| Buner | Daggar | 34.5107 | 72.4835 | Semi-urban agriculture and livestock rearing |
| Swat | Islampur | 34.7211 | 72.3652 | Riverine agriculture and grazing |
| Swat | Bagh | 35.0537 | 72.4734 | Mixed agriculture with orchard farming |
| Swat | Tangobanda | 34.6088 | 72.5984 | Mountain agriculture and seasonal grazing |
| Swat | Kokarai | 34.7387 | 72.4251 | Mixed valley agriculture and pastureland |
| Swat | Guligram | 34.7330 | 72.3330 | Upland grazing and subsistence farming |
| Swat | Bara Bandai | 34.8245 | 72.3947 | Highland grazing with sparse cultivation |
