# Supplementary material for: Meta-transcriptomic characterization reveals viral species with zoonotic potential in Rhipicephalus microplus and Haemaphysalis bispinosa ticks in Pakistan
Source: Vet Res. 2026 Mar 26;57:56. doi: 10.1186/s13567-026-01739-5 (PMC13107747; doi:10.1186/s13567-026-01739-5)
Supplement: Supplementary file 2 — Additional file 2. Mapping Statistics for putative novel viruses identified in this study. [file 13567_2026_1739_MOESM2_ESM.doc]

**Additional file 2.** Mapping Statistics for putative novel viruses identified in this study.

| Virus species | Library ID | Accession | ORF | Length (bp) | Mapped Reads | Mean reads depth | Coverage (%) |
| --- | --- | --- | --- | --- | --- | --- | --- |
| Pakistan luteovirus | PAK-04 | PV129945 | RdRP | 1521 | 2709 | 5795.04 | 94.89 |
| Pakistan luteovirus | PAK-10 | PV167496 | RdRP | 1518 | 2184 | 7.09 | 89.97 |
| Pakistan microplus virus | PAK-16 | PV129938 | RdRP | 3912 | 4050 | 102.64 | 99.93 |
| Pakistan microplus virus | PAK-16 | PV129939 | VP2 | 2841 | 2724 | 148.91 | 100 |
| Pakistan microplus virus | PAK-16 | PV129940 | VP3 | 2317 | 2069 | 134.36 | 98.34 |
| Pakistan microplus virus | PAK-16 | PV129941 | VP4 | 2135 | 2045 | 92.04 | 100 |
| Pakistan microplus virus | PAK-16 | PV129942 | VP5 | 1954 | 1629 | 188.6 | 100 |
| Pakistan microplus virus | PAK-16 | PV167511 | NS1 | 1726 | 1662 | 164.63 | 97.54 |
| Pakistan microplus virus | PAK-16 | PV167512 | NS2 | 1502 | 1492 | 172.84 | 100 |
| Pakistan microplus virus | PAK-16 | PV167513 | VP7 | 1384 | 1275 | 311.47 | 100 |
| Pakistan microplus virus | PAK-16 | PV167514 | VP6 | 1212 | 718 | 310.19 | 100 |
| Pakistan microplus virus | PAK-16 | PV167515 | NS3 | 1102 | 775 | 193.1 | 91.72 |
