# Supplementary material for: Meta-transcriptomic characterization reveals viral species with zoonotic potential in Rhipicephalus microplus and Haemaphysalis bispinosa ticks in Pakistan
Source: Vet Res. 2026 Mar 26;57:56. doi: 10.1186/s13567-026-01739-5 (PMC13107747; doi:10.1186/s13567-026-01739-5)
Supplement: Supplementary file 3 — Additional file 3. Basic information of each library for sequencing in this study. [file 13567_2026_1739_MOESM3_ESM.docx]

**Additional file 3.** Basic information of each library for sequencing in this study

| Tick species | District | Library ID | Sex | Host | host-derived (%) | Clean reads (paired-end) | Viral reads (%) | Ticks per library |
| --- | --- | --- | --- | --- | --- | --- | --- | --- |
| *R. microplus* | Swabi | PAK-01 | Female | cow | 68.09 | 12,324,552 | 0.03 | 5 |
| *R. microplus* | Swabi | PAK-02 | Female | cow | 58 | 16,590,908 | 0.26 | 4 |
| *R. microplus* | Swabi | PAK-03 | Male | cow | 0.14 | 34,368,100 | 0.11 | 7 |
| *R. microplus* | Swabi | PAK-04 | Male | cow | 26.82 | 29,986,207 | 0.01 | 8 |
| *H. bispinosa* | Swabi | PAK-05 | Female | sheep | 73.53 | 9,978,557 | 0.06 | 6 |
| *H. bispinosa* | Swabi | PAK-06 | Female | sheep | 73.94 | 9,473,063 | 0.15 | 8 |
| *H. bispinosa* | Swabi | PAK-07 | Male | sheep | 75.69 | 9,599,681 | 0.07 | 10 |
| *H. bispinosa* | Swabi | PAK-08 | Male | sheep | 1.98 | 30,855,940 | 0.12 | 9 |
| *R. microplus* | Buner | PAK-09 | Female | cow | 85.42 | 6,141,674 | 0.01 | 9 |
| *R. microplus* | Buner | PAK-10 | Female | cow | 26.51 | 27,996,235 | 0.1 | 8 |
| *R. microplus* | Buner | PAK-11 | Male | cow | 0.07 | 34,293,794 | 0.11 | 6 |
| *R. microplus* | Buner | PAK-12 | Female | sheep | 81.04 | 6,355,235 | 0.09 | 5 |
| *R. microplus* | Buner | PAK-13 | Female | sheep | 88.06 | 3,596,708 | 0.17 | 5 |
| *R. microplus* | Buner | PAK-14 | Male | sheep | 84.71 | 5,490,626 | 0.59 | 7 |
| *R. microplus* | Buner | PAK-15 | Male | sheep | 1.32 | 34,172,398 | 0.12 | 8 |
| *R. microplus* | Swat | PAK-16 | Female | cow | 87.87 | 4,606,279 | 0.1 | 5 |
| *R. microplus* | Swat | PAK-17 | Female | cow | 52.61 | 14,440,686 | 0.05 | 7 |
| *R. microplus* | Swat | PAK-19 | Male | cow | 67.71 | 11,063,939 | 0.08 | 6 |
| *R. microplus* | Swat | PAK-20 | Female | cow | 77.73 | 6,720,705 | 0.25 | 9 |
| *R. microplus* | Swat | PAK-21 | Female | cow | 84.93 | 4,449,811 | 0.16 | 7 |
| *R. microplus* | Swat | PAK-22 | Male | cow | 2.78 | 33,203,140 | 0.14 | 6 |
| *R. microplus* | Swat | PAK-23 | Male | cow | 73.63 | 9,944,283 | 17 | 8 |
| *R. microplus* | Swat | PAK-24 | Male | cow | 8.95 | 31,062,389 | 0.13 | 9 |
