# Supplementary material for: Meta-transcriptomic characterization reveals viral species with zoonotic potential in Rhipicephalus microplus and Haemaphysalis bispinosa ticks in Pakistan
Source: Vet Res. 2026 Mar 26;57:56. doi: 10.1186/s13567-026-01739-5 (PMC13107747; doi:10.1186/s13567-026-01739-5)
Supplement: Supplementary file 5 — Additional file 5. Viral sequences of this study deposited in GenBank. [file 13567_2026_1739_MOESM5_ESM.doc]

**Additional file 5.** Viral sequences of this study deposited in GenBank

| Accession no. | Sample ID | Species |
| --- | --- | --- |
| PV129932 | PAK-07 | Hepelivirales sp. |
| PV129943 | PAK-06 | Hubei sobemo-like virus 15 |
| PV129944 | PAK-06 | Hubei sobemo-like virus 15 |
| PV129921 | PAK-14 | Mogiana tick virus |
| PV129922 | PAK-14 | Mogiana tick virus |
| PV129923 | PAK-14 | Mogiana tick virus |
| PV129924 | PAK-16 | Mogiana tick virus |
| PV129925 | PAK-16 | Mogiana tick virus |
| PV129926 | PAK-22 | Mogiana tick virus |
| PV129927 | PAK-23 | Mogiana tick virus |
| PV129928 | PAK-23 | Mogiana tick virus |
| PV129929 | PAK-23 | Mogiana tick virus |
| PV129930 | PAK-23 | Mogiana tick virus |
| PV129931 | PAK-14 | Mogiana tick virus |
| PV167497 | PAK-16 | Mogiana tick virus |
| PV167498 | PAK-16 | Mogiana tick virus |
| PV167499 | PAK-21 | Mogiana tick virus |
| PV167500 | PAK-20 | Mogiana tick virus |
| PV167501 | PAK-20 | Mogiana tick virus |
| PV167502 | PAK-20 | Mogiana tick virus |
| PV167503 | PAK-21 | Mogiana tick virus |
| PV167504 | PAK-21 | Mogiana tick virus |
| PV167505 | PAK-21 | Mogiana tick virus |
| PV167506 | PAK-22 | Mogiana tick virus |
| PV129933 | PAK-12 | *Uukuvirus lihanense* |
| PV129934 | PAK-13 | *Uukuvirus lihanense* |
| PV129935 | PAK-21 | *Uukuvirus lihanense* |
| PV129936 | PAK-23 | *Uukuvirus lihanense* |
| PV129937 | PAK-14 | *Uukuvirus lihanense* |
| PV167507 | PAK-14 | *Uukuvirus lihanense* |
| PV167508 | PAK-21 | *Uukuvirus lihanense* |
| PV167509 | PAK-23 | *Uukuvirus lihanense* |
| PV129945 | PAK-04 | Pakistan luteovirus |
| PV167496 | PAK-10 | Pakistan luteovirus |
| PV129938 | PAK-16 | Pakistan microplus virus |
| PV129939 | PAK-16 | Pakistan microplus virus |
| PV129940 | PAK-16 | Pakistan microplus virus |
| PV129941 | PAK-16 | Pakistan microplus virus |
| PV129942 | PAK-16 | Pakistan microplus virus |
| PV167511 | PAK-16 | Pakistan microplus virus |
| PV167512 | PAK-16 | Pakistan microplus virus |
| PV167513 | PAK-16 | Pakistan microplus virus |
| PV167514 | PAK-16 | Pakistan microplus virus |
| PV167515 | PAK-16 | Pakistan microplus virus |
| PV167510 | PAK-20 | Rhabdoviridae sp. |
| PV167516 | PAK-01 | *Tobacco mosaic virus* |
| PV167517 | PAK-12 | Totiviridae sp. |
| PV167518 | PAK-16 | Totiviridae sp. |
| PV167519 | PAK-23 | Totiviridae sp. |
| PV129914 | PAK-12 | *Wuhan mivirus* |
| PV129915 | PAK-13 | *Wuhan mivirus* |
| PV129916 | PAK-16 | *Wuhan mivirus* |
| PV129917 | PAK-20 | *Wuhan mivirus* |
| PV129918 | PAK-21 | *Wuhan mivirus* |
| PV129919 | PAK-23 | *Wuhan mivirus* |
| PV129920 | PAK-02 | *Wuhan mivirus* |
