# Supplementary figures and images for: Meta-transcriptomic characterization reveals viral species with zoonotic potential in Rhipicephalus microplus and Haemaphysalis bispinosa ticks in Pakistan
Source: Vet Res. 2026 Mar 26;57:56. doi: 10.1186/s13567-026-01739-5 (PMC13107747; doi:10.1186/s13567-026-01739-5)

## VP2

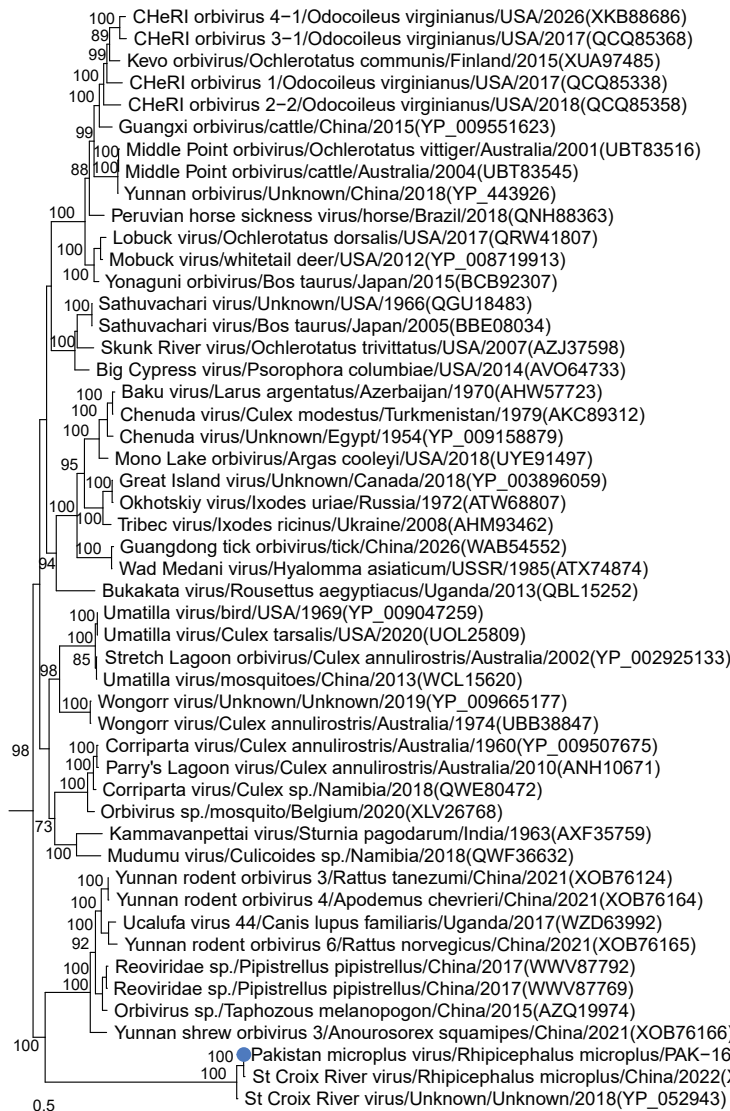

## VP6

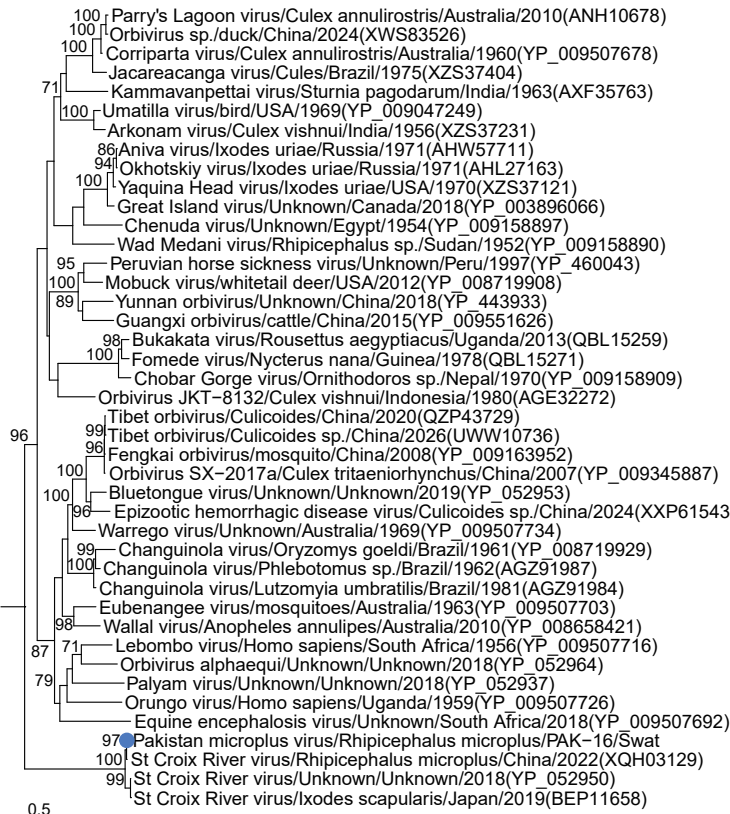

## VP3

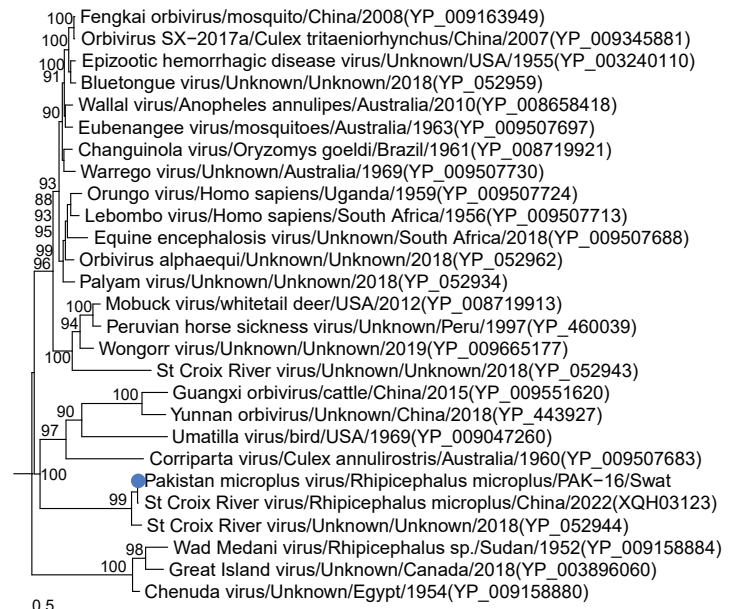

## VP4

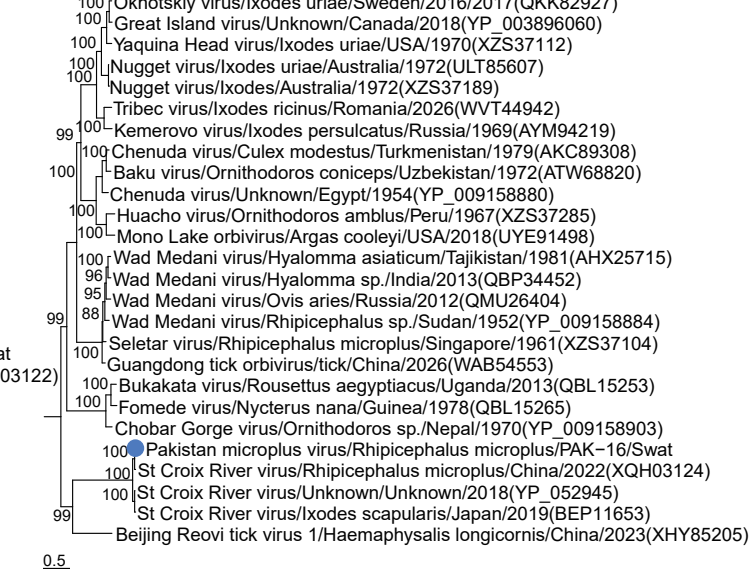

## VP5

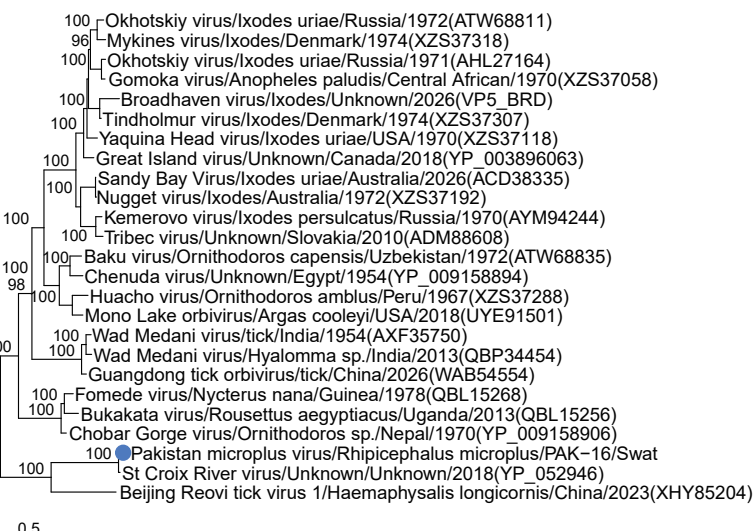

Supplement: Supplementary file 6 — Additional file 6. Phylogenetic tree of orbivirus strains showing their genetic relationships across various hosts and region. [file 13567_2026_1739_MOESM6_ESM.pdf]

## VP7

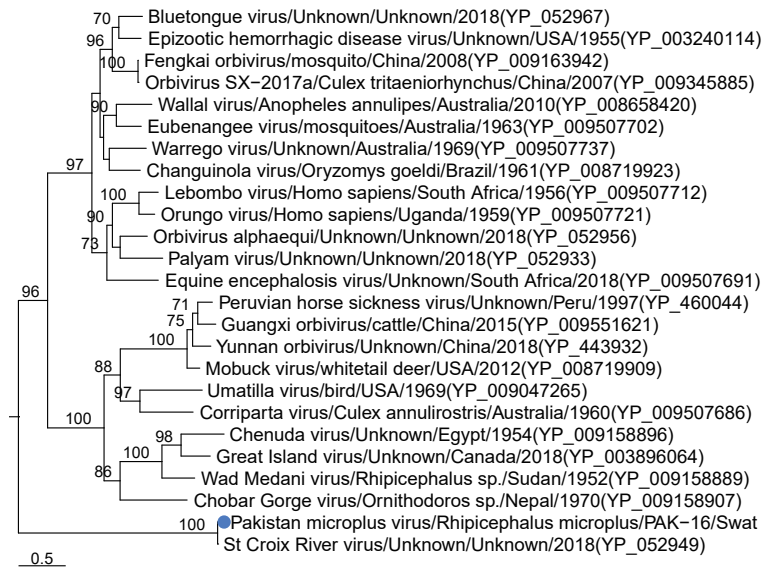

## NS2

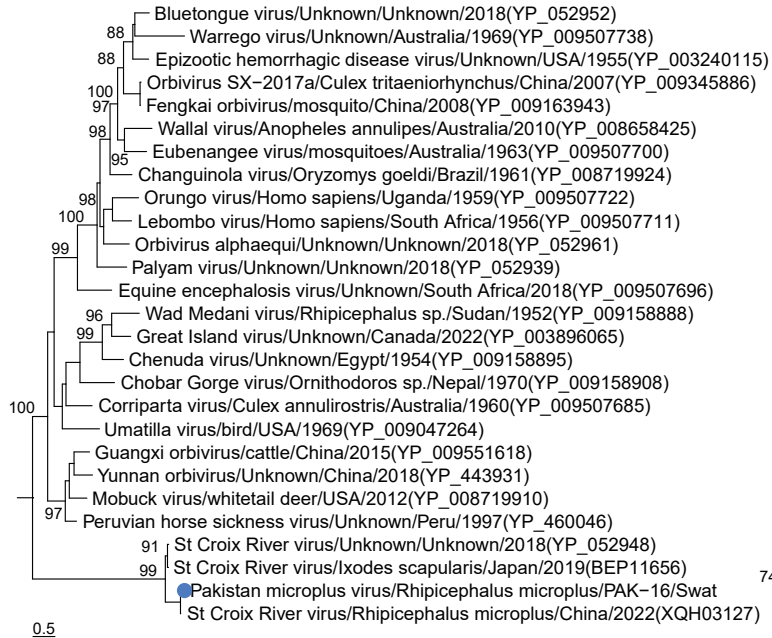

## NS3

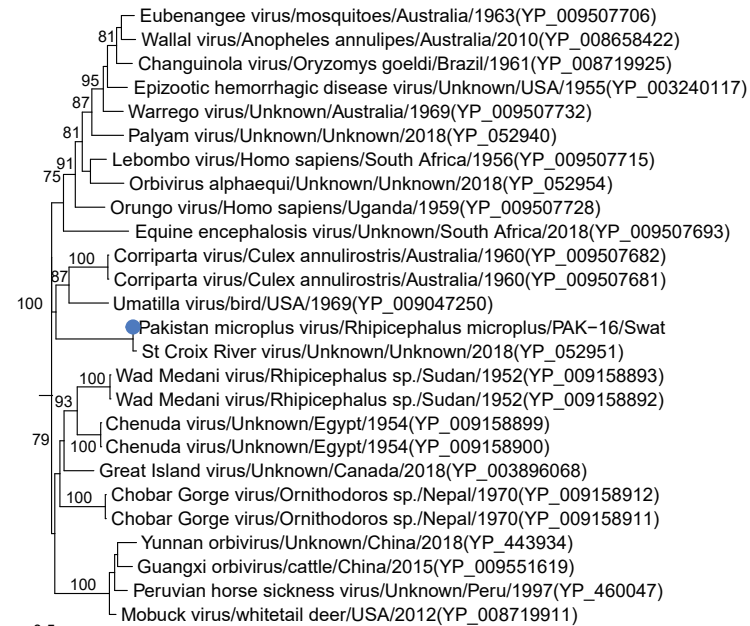

## NS1

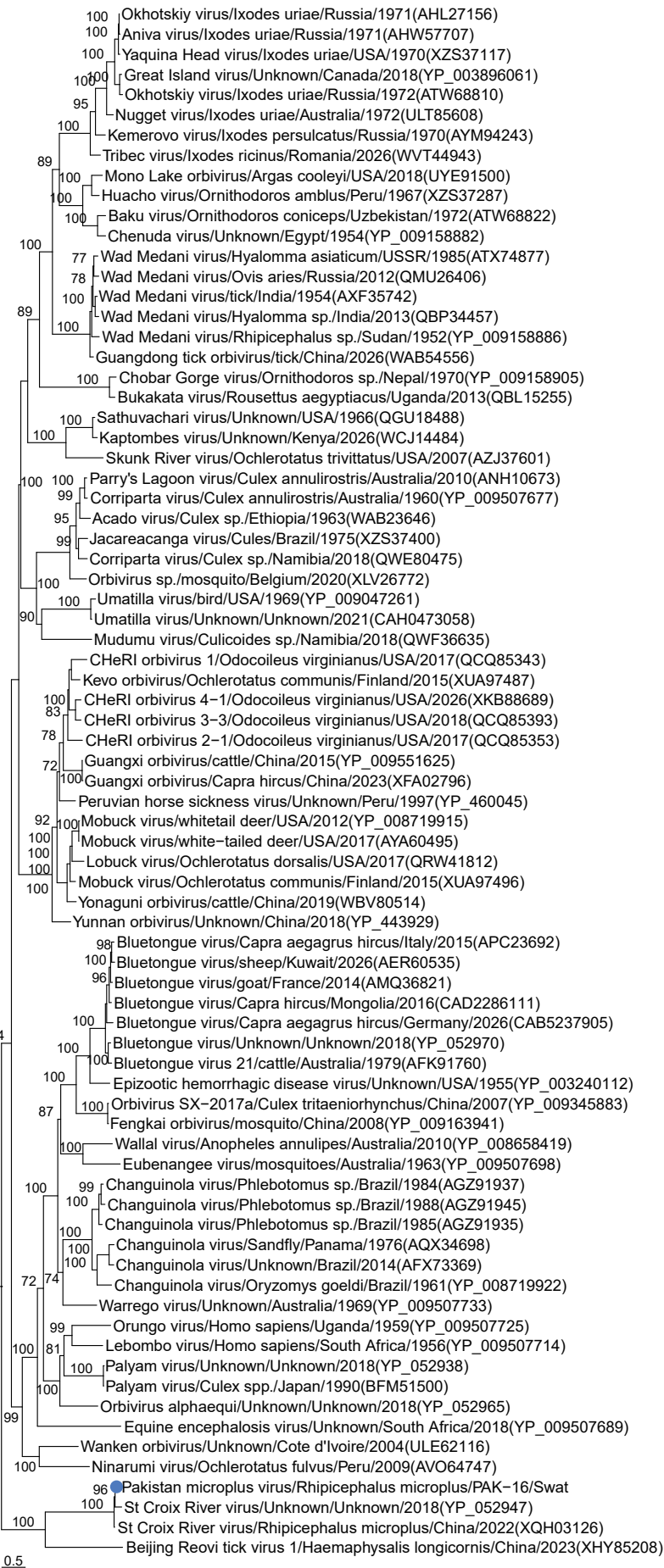

Supplement: Supplementary file 7 — Additional file 7. Phylogenetic tree of orbivirus strains based on VP7, NS1, NS2, and NS3 protein sequences, illustrating their molecular relationships. [file 13567_2026_1739_MOESM7_ESM.pdf]
